# Luminophore Configuration and Concentration-Dependent Optoelectronic Characteristics of a Quantum Dot-Embedded DNA Hybrid Thin film

**DOI:** 10.1038/s41598-017-11797-7

**Published:** 2017-09-14

**Authors:** Velu Arasu, Sreekantha Reddy Dugasani, Mallikarjuna Reddy Kesama, Ho Kyoon Chung, Sung Ha Park

**Affiliations:** 1 Sungkyunkwan Advanced Institute of Nanotechnology (SAINT), Sungkyunkwan University, Suwon, 16419 Korea; 2 Department of Physics, Sungkyunkwan University, Suwon, 16419 Korea

## Abstract

To be useful in optoelectronic devices and sensors, a platform comprising stable fluorescence materials is essential. Here we constructed quantum dots (QDs) embedded DNA thin films which aims for stable fluorescence through the stabilization of QDs in the high aspect ratio salmon DNA (SDNA) matrix. Also for maximum luminescence, different concentration and configurations of core- and core/alloy/shell-type QDs were embedded within SDNA. The QD-SDNA thin films were constructed by drop-casting and investigated their optoelectronic properties. The infrared, UV-visible and photoluminescence (PL) spectroscopies confirm the embedment of QDs in the SDNA matrix. Absolute PL quantum yield of the QD-SDNA thin film shows the ~70% boost due to SDNA matrix compared to QDs alone in aqueous phase. The linear increase of PL photon counts from few to order of 5 while increasing [QD] reveals the non-aggregation of QDs within SDNA matrix. These systematic studies on the QD structure, absorbance, and concentration- and thickness-dependent optoelectronic characteristics demonstrate the novel properties of the QD-SDNA thin film. Consequently, the SDNA thin films were suggested to utilize for the generalised optical environments, which has the potential as a matrix for light conversion and harvesting nano-bio material as well as for super resolution bioimaging- and biophotonics-based sensors.

## Introduction

Deoxyribonucleic acid (DNA) molecules are commercially available at relatively low cost and have unique polymer characteristics, and so have potential as functional biomaterials in various fields, such as biophotonics, bioelectronics, and biosensors. A thin film made of DNA shows low optical loss and high transparency^1^. These unique and superior optoelectronic characteristics of DNA are useful for solid state devices as a charge transport layer in functional devices and a host medium for luminophores in photonics. Cationic-modified DNA material has been used as a selective charge transport layer in light-emitting diodes (LEDs)^2^, organic light-emitting diodes (OLEDs)^3, 4^ and organic field-effect transistor devices (OFETs)^5, 6^. DNA materials have been used as hosts of fluorescent dye^7^ and metal ions^8^ by doping and resulted in amplified emissions in lasing^9–11^, nonlinear behaviour in optics^12^ and also in wave-guiding applications^13^.

Although DNA itself has important physical, chemical, and biological characteristics, DNA as a template for hosting functionalised materials^14, 15^ such as nanoparticles, di- and tri-valent ions, fluorescent dyes, carbon-based materials, proteins and drugs might be superior in terms of specific functionality enhancement and reliable output due to its specific binding affinity to the functionalised guest materials and ability to be modified. The presence of the hydrogen bonding by DNA molecules amplifies the fluorescence emissions of a given luminophore^16^. Among the available luminophores, DNA molecules with fluorescent dyes and metal ions have been used to study optical characteristics. However, other fluorescence materials; *i.e*. quantum dots (QDs) which have unique optoelectric characteristics in terms of stable fluorescence, large Stoke shift, quantum confinement and semiconducting nature, warrant investigation.

QDs are fluorescent semiconducting nanoparticles with confined electrons at a scale of a few nanometres that exhibit a quantum size effect^17, 18^. QDs enable mimicking of natural biomaterials and so could replace conventional electronics and photonics materials^19–21^. In biological research, QDs have potential as biomarkers for site-specific drug delivery and medical imaging^22^. For use in biological applications, QDs must be soluble in aqueous liquids with high colloidal stability, which requires an optimised surface ligand exchange process with appropriate surface ligands^23^. QDs and DNA materials have different optoelectronic characteristics in different phases; *i.e*. the colloidal and condensed matter (thin film) phases. Although conjugation of QDs to DNA in the colloidal phase has been reported to facilitate florescence imaging and sensing applications^24, 25^, DNA and QDs in the condensed matter phase (in the form of a thin film) has been the subject of few studies.

To be useful ‒ *e.g*. overcome concentration-dependent luminescence quenching^26^, performance inconsistency, non-biocompatibility, and structural and photo instability ‒ in optoelectronic devices and sensors, a stable platform (production of thin films with easily tunable thickness) using stable fluorescence materials (efficient dispersibility in a given platform) is essential. Here we developed a natural salmon DNA (SDNA) thin film (as a stable template) with QDs (photo stable inorganic luminophores) dispersed in aqueous and organic phases and investigated the optoelectronic characteristics of QD-embedded SDNA thin films. Two QD luminophore configurations ‒ core-type QD (QDC) and core/alloy/shell-type QD (QDA) ‒ were synthesised by the one-pot hot-injection method. As-synthesised QD surface were replaced by hydrophilic ligands (for aqueous compatibility). Here the core/shell configuration was adopted in order to maintain the photoluminescence quantum yield (PLQY) and avoid structural instability in an aqueous phase. An appropriate amount of surface functionalised QDs was mixed with SDNA molecules in an aqueous phase to fabricate thickness-controllable QD-embedded SDNA thin films by a drop-casting method.

The synthesised QDs were evaluated by high-resolution transmission electron microscopy (HRTEM), scanning transmission electron microscopy (STEM), high-angle annular dark-field (HAADF) elemental-mapping (for verification of the internal structures of QDs), atomic force microscopy (AFM) (for visualisation of the topological profile), Fourier transform infrared (FTIR) spectroscopy (for confirmation of the presence of ligands on QDs), and UV-vis and PL spectroscopies (for evaluation of their optical characteristics). We also investigated the QD-embedded SDNA thin films by the FTIR spectrum to confirm the QD and SDNA configuration, UV-vis and PL spectra to assess their photonic characteristics and current‒voltage (*I‒V*) measurements to evaluate their electrical properties.

## Results

### Internal structure and topological characteristics of synthesised and surface-modified QDs

A QD is a relatively more stable fluorescent material than the conventionally available fluorescent dye molecules. Emission of QDs is tunable across the entire visible range by means of bandgap engineering. Among the QDs (normally dispersed in an organic phase), cadmium-selinium (CdSe) binary QDs (core type, QDC) have a high PLQY up to 85%^27^. However core-type QDs in an aqueous phase exhibit a fivefold reduction in PLQY due to the surface modification required for stabilisation in water^28^. The core/alloy/shell configuration of QD (QDA) was synthesised by one-pot method, and exhibited enhanced structural stability compared to core/shell-type QDs (multi-pot preparation required and relatively low structural stability due to a high lattice mismatch)^29^.

**Figure 1:**
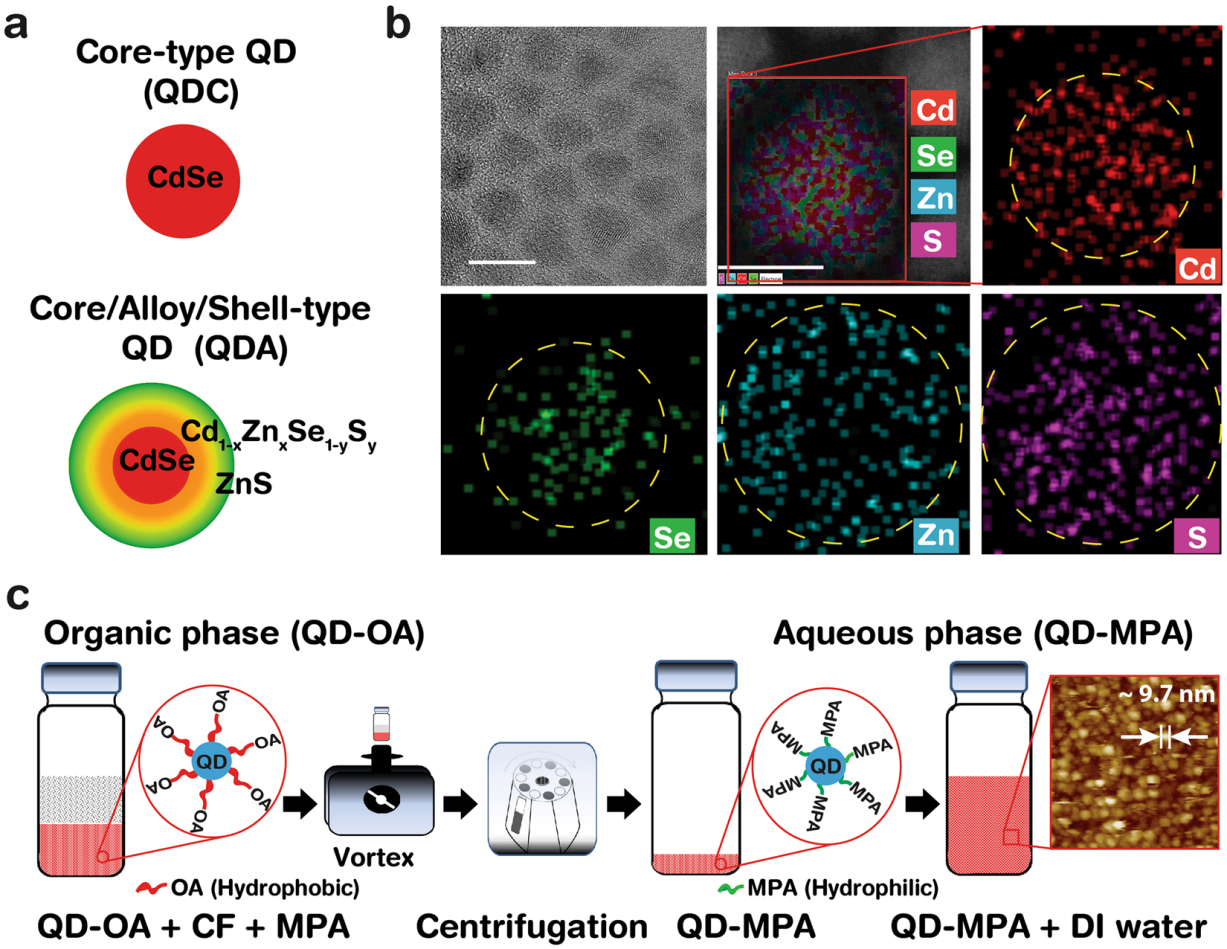
Schematics of QDC(A), HRTEM/STEM/HAADF elemental mapping, and ligand exchange of QDs. (**a**) Schematics of internal structures of CdSe core-type QDs (QDC) and CdSe/Cd_1−x_Zn_x_Se_1−y_S_y_/ZnS core/alloy/shell-type QD (QDA). (**b**) High-resolution transmission electron microscope (HRTEM) (with scale bar, 10 nm), scanning transmission electron microscope (STEM) (scale bar, 5 nm) and high-angle annular dark-field (HAADF) (scale bar, 5 nm) elemental-mapping images of QDAs. The internal elemental distribution of QDAs confirms core/alloy/shell structure formation. (**c**) A schematic of ligand exchange process of QD dispersed in chloroform (CF) with oleic acid (OA) ligand which was replaced by 3-mercaptopropionic acid (MPA) ligand through the mass-action principle. The QD-MPAs were readily dispersed in deionised (DI) water. A representative AFM image (far right) of as-surface functionalized QDA-MPA (scan size, 200 × 200 nm^2^) obtained by the fluid-tapping mode. Average QDA-MPA hydrodynamic-diameter was 9.68 ± 0.2 nm.

QD synthesis was shown in Supplementary Figure S1, and discussed in the methods section. QDC and QDA structures were formed in the absence and presence of Zn and S, respectively. Schematic diagrams of QDC and QDA are shown in Fig. 1a. The TEM and STEM images (Fig. 1b) show a QDA size of ~7 nm with moderate distribution, and HAADF images show the single-particle internal elemental distribution and mapping. The single particle elemental mapping of Cd, Se, Zn, and S shows that the alloy QD configurations (QDA) are in agreement with the energy dispersive spectrum (EDS) and quantitative atomic and weight percentage data (Supplementary Figure S2).

Figure 1c shows QD surface modification by ligand exchange process (discussed in detail in the Methods section) in which the pre-synthesised oleic acid (OA, C_18_H_34_O_2_) capped hydrophobic ligands are replaced by 3-mercaptopropionic acid (MPA, C_3_H_6_O_2_S) capped hydrophilic ligands, to facilitate homogeneous dispersion of the QDs in an aqueous phase. The ligand exchange method used was simple and efficient; mixing of MPA (as a phase-transfer agent) with target QDs. A representative AFM image of QDA in an aqueous phase is shown in Fig. 1c. The average hydrodynamic diameter (obtained in a colloidal phase) of QDA was 9.68 ± 0.2 nm, which was slightly larger than the TEM value (obtained in a dry phase) due to inclusion of surface ligands in the aqueous phase (Supplementary Figure S3).

### Fourier transform infrared, UV-vis, and photoluminescence characteristics of a QDC and a QDA

**Figure 2:**
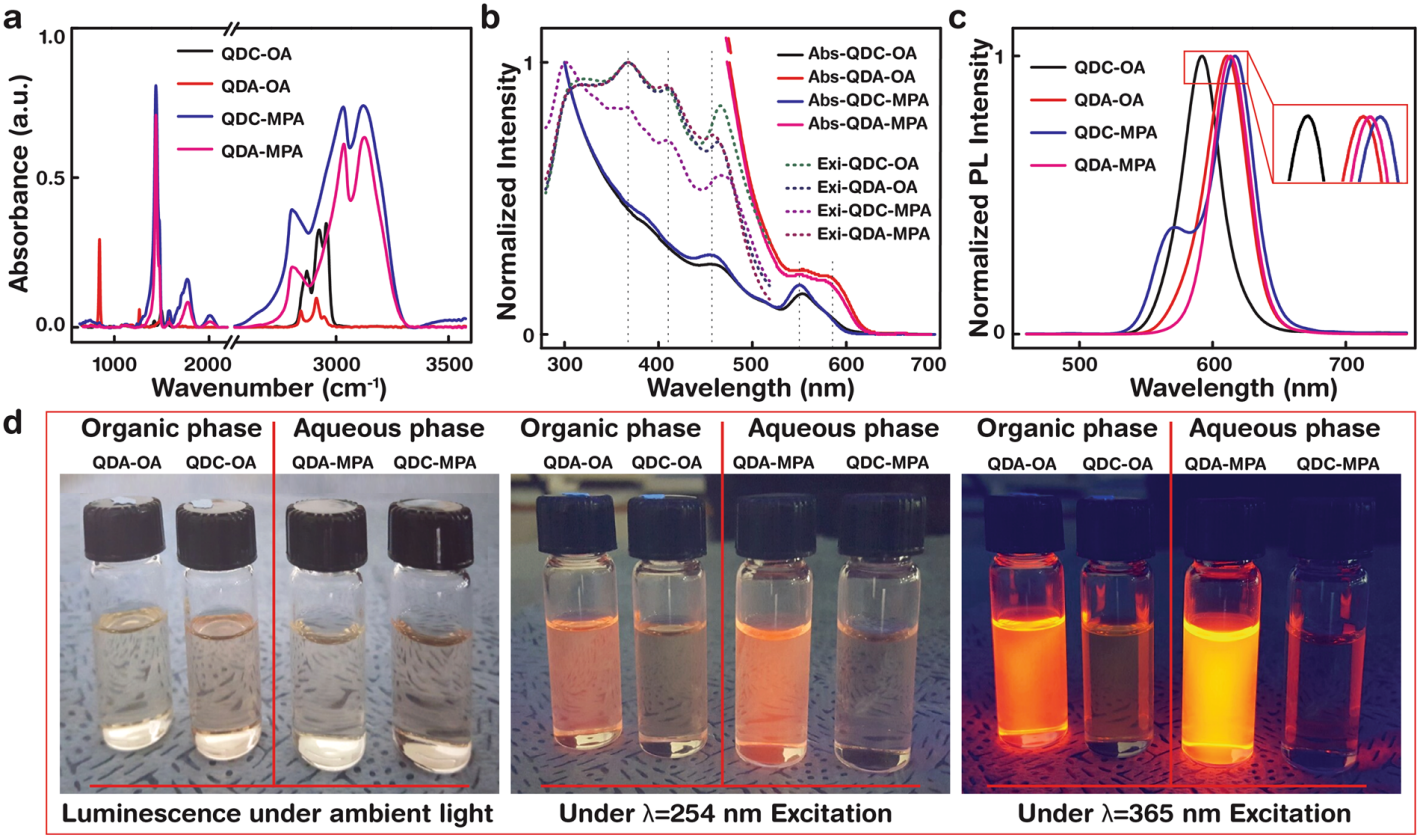
Optical characteristics of QD-OA and QD-MPA samples. QDCs and QDAs were dispersed in chloroform (organic) with oleic acid (OA) ligand (QDC-OA, QDA-OA) and dispersed in DI water (aqueous) with 3-mercaptopropionic acid (MPA) ligand (QDC-MPA, QDA-MPA), respectively. (**a**) Fourier transform infrared (FTIR) spectra of colloidal QD-OAs and QD-MPAs. The absorbance bands found at around λ^−1^ = 2900, 1400 and 1750 cm^−1^ indicated C-H stretching vibrations of OA ligand attachment to QD-OA, the carboxylate symmetric and asymmetric stretching vibrations of MPA ligand attachment to QD-MPA, respectively. (**b**) Normalised UV-vis absorption (Abs., solid lines) and photoluminescence excitation (Exi., dotted lines) spectra of colloidal QD-OAs and QD-MPAs. The first and second absorption peaks were found at λ = 556, 588 nm and at λ = 456, 560 nm for the colloidal QD-OAs and QD-MPAs, respectively (vertical dotted lines). Photoluminescence excitation spectra maximum was found at λ = 370 nm for both QD-OAs and QD-MPAs. (**c**) Fluorescence emission of QDCs and QDAs were obtained by exciting at λ = 370 nm. Organic QDC-OA and QDA-OA showed emissions at λ = 589 and 608 nm, respectively. Due to ligand exchange, aqueous QDC-MPA and QDA-MPA showed red-shifted emissions at λ = 614 and 611 nm, respectively (overlapped peaks are magnified for clarity). (**d**) Luminescence photographs of colloidal QD-OAs and QD-MPAs. Luminescence under the excitation wavelengths; *i.e*. ambient light, 254 nm and 365 nm revealed excitation-dependent emission characteristics.

The synthesised QDs were analysed by FTIR spectrometry (see the Methods section) to confirm ligand attachment on the QD surface. QDs capped with an OA ligand (QD-OA) and MPA (QD-MPA) are compatible with chloroform and deionized (DI) water solvents, respectively. Figure 2a shows the FTIR absorbance peaks of QDC-OA (organic), QDA-OA (organic), QDC-MPA (aqueous), and QDA-MPA (aqueous). The absorbance characteristic peak at 2,900 cm^−1^ is attributable to C-H stretching vibration of OA ligand attached to QDC-OA and QDA-OA; similarly, the absorbance characteristic peaks at 1,400 and 1,750 cm^−1^ indicate the carboxylate symmetric and asymmetric stretching vibrations of MPA ligand attached to QDC-MPA and QDA-MPA. The peak assignments are in good agreement with a previous report (Supplementary Table S1)^30^. Figure 2b shows the UV-vis absorption (Abs., solid line) and PL excitation (Exi., dotted) spectra of colloidal QDC-OA, QDA-OA, QDC-MPA, and QDA-MPA. Solid lines represent the first (second) excitonic peak maximum at 556 nm (456) and 588 nm (560) for QDC-OA and QDA-OA, respectively. QDC-MPA and QDA-MPA dispersed in an aqueous phase are blue-shifted by ~4 nm due to shortening of the ligand length on the QD surface by the ligand exchange process ‒ a longer OA ligand (hydrophobic) was replaced by a shorter MPA (hydrophilic) to enhance the aqueous dispersibility. The dotted line represents the PL excitation of QDC and QDA samples; the overall PL excitation spectra maximum was observed at 370 nm. The second and higher excitation peaks of absorbance and PL excitation peaks have some resemblance (shown by vertical dotted lines in Fig. 2b). However the PL excitation peaks have excitation maximum at ~370 nm and falls but absorbance peaks keep heading towards maximum without fall at shorter wavelength.

Fluorescence emission of colloidal QDC and QDA were obtained by PL spectroscopy (discussed in detail in the Methods section), exciting at 370 nm; fluorescence emissions were found at 590 and 608 nm for QDC-OA (absolute PLQY: 7%) and QDA-OA (PLQY: 61%) dispersed in an organic solvent, respectively (Fig. 2c). QDC-MPA (PLQY: 2%) and QDA-MPA (PLQY: 13%) dispersed in an aqueous medium showed red-shifted emissions from 590 to 614 and 608 to 611 nm, respectively due to the ligand-exchange process. The normalized PL spectra of QDs show smooth curves with full-width at half-maximum (FWHM) of about 35 and 38 nm for QDC-OA and QDA-OA, respectively. Also, the ligand-exchange process alters the PL FWHM from 35 to 39 nm (increased) and 38 to 35 nm (decreased) for QDC-MPA and QDA-MPA, respectively; these narrow PL emission characteristics of QDs would facilitate sensing applications (*e.g*. bio-labelling and diagnostic). Figure 2d shows the luminescence of organic (QDC-OA, QDA-OA) and aqueous (QDC-MPA, QDA-MPA) QDs; luminescence under UV light with excitation wavelengths of 254 and 365 nm clearly shows excitation wavelength-dependent emission characteristics (compared to ambient light), which visually confirm the importance of the optical characteristics of the synthesised QD (*i.e*. QDC and QDA).

### Fourier transform infrared absorbance and luminescence analysis of QD-embedded SDNA thin films

**Figure 3:**
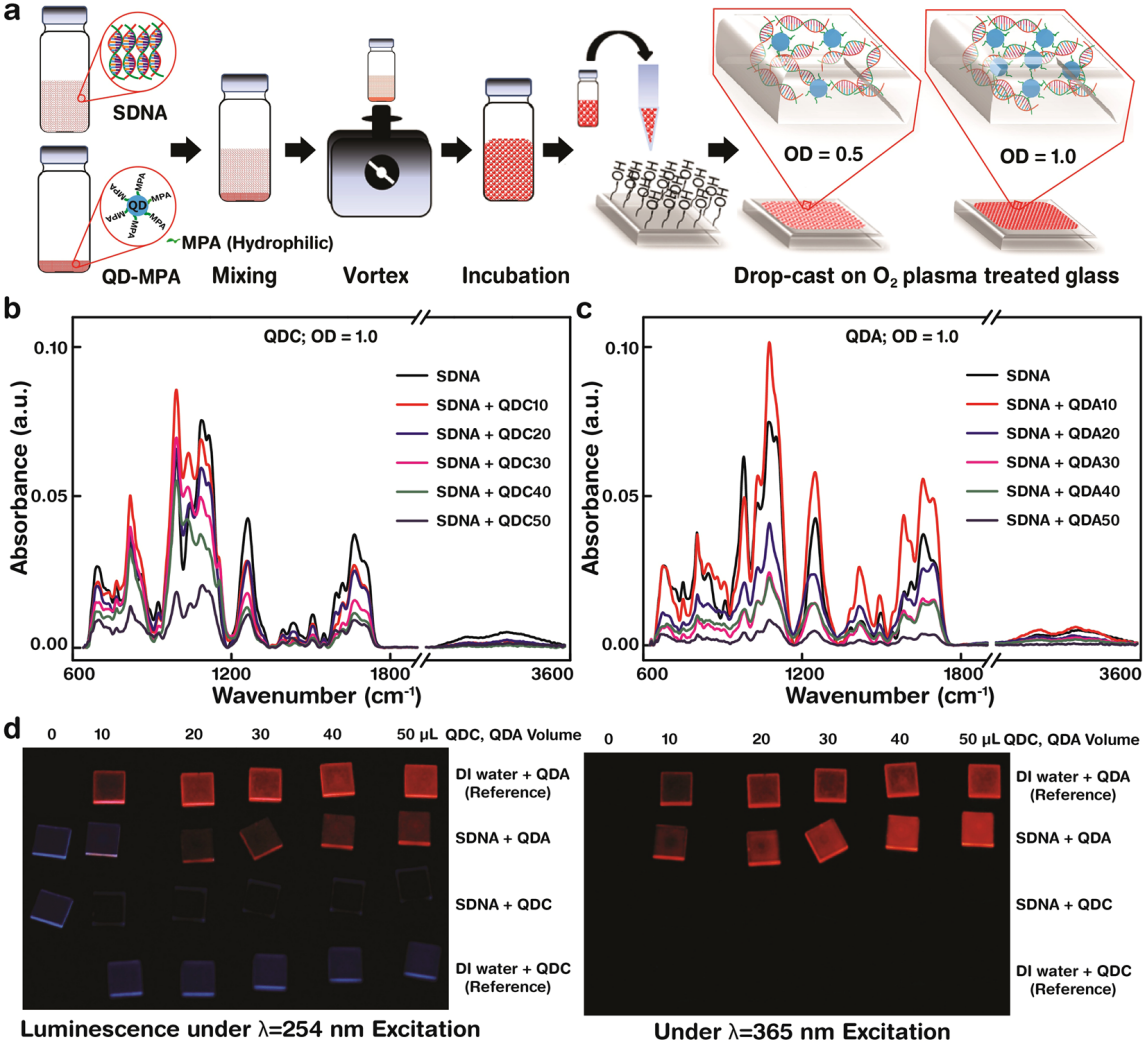
The fabrication, Fourier transform infrared absorbance and luminescence of QD-embedded SDNA thin films. (**a**) Fabrication schematic of a QD-embedded salmon DNA (SDNA) thin film. The thin film was fabricated by mixing individual colloidal QD-MPA (hydrophilic) of two concentrations (*i.e*. OD 0.5 and 1.0) and SDNA (dispersed in DI water at 1 wt.-%) followed by drop-casting on an O_2_ plasma-treated (hydrophilic) glass substrate. (**b**,**c**) Fourier transform infrared (FTIR) absorbance spectra of SDNA thin films with QDCs and QDAs (OD 1.0). The decrease of absorbance peaks with increasing QD volume (here QDC(A)10 was assigned for 10 µL of QDC(A)) at a given SDNA volume of 160 µL. Similarly 20, 30, 40, and 50 µL of QDC(A) are labelled QDC(A)20, QDC(A)30, QDC(A)40, and QDC(A)50, respectively, in the legend) indicated no chemical interaction between the QDs and the DNA molecules. Consequently, the SDNA thin film acts as an efficient host for QD luminophores. (**d**) Luminescence of SDNA thin films with various concentrations of QDCs and QDAs (controlled by volume). The luminescences under excitation wavelengths of 254 nm (UV light reflections were appeared as blue) and 365 nm showed significant excitation-dependent (red) emission characteristics of QDAs as indicated by the different optical emissions.

The volumetric ratio of SDNA to QD (SDNA:QD) was varied for a reasonable amount of QD (hereon all the QD’s samples were dispersed in DI water which have MPA ligand) embedding condition within the condensed thin film. By using one weight-percentage (1 wt.-%) of SDNA and 1.0 optical density (OD) of QDs (which correspond to first excitation peak’s absorbance value of QD, obtained by UV-vis spectrophotometer), we prepared a homogeneous 20 µL QD-embedded SDNA colloidal mixture of six SDNA:QD ratios of 16:0 (20 µL of SDNA without QDs, served as a reference), 16:1 (18.8 µL of SDNA with 1.2 µL of QDs, named QD10), 16:2 (17.8 µL SDNA with 2.2 µL QDs, QD20), 16:3 (16.8 µL SDNA with 3.2 µL QDs, QD30), 16:4 (16.0 µL SDNA with 4.0 µL QDs, QD40), and 16:5 (15.2 µL SDNA with 4.8 µL QDs, QD50). The mixtures were dropped on an O_2_ plasma-treated quartz substrate. The fabrication and sample preparation procedures are described in Fig. 3a, the Methods section, and Supplementary Table S2.

To evaluate the optical characteristics of a QD and SDNA mixture, the absorbance of SDNA thin films with different concentrations (controlled by the volume of QDs) of QDCs and QDAs was determined (Fig. 3b,c). The absorbance peaks that indicate the binding environment and chemical functional groups of DNA molecules and the corresponding characteristic peaks were assigned accordingly (Supplementary Table S4)^15^. Based on the absorbance measurements, strong (weak) characteristic peaks were found at 780, 960, 1010, 1055, 1083, 1230, and 1652 cm^−1^ (828, 895, 1372, 1416, 1488, 1604, 1693, and 3357 cm^−1^). The absorbance bands were divided into three regions: 3600–3000 cm^−1^ for water OH stretching, 1800–1300 cm^−1^ for nucleotide bases, and 1250–600 cm^−1^ for sugar and phosphate groups. The decrement of absorbance intensities was observed for both QDC- and QDA-embedded SDNA thin films because of the increment of QDs (caused decrement of SDNA concentration) within the SDNA matrix. Interestingly, either an appearance or a disappearance of a certain absorbance peak was not observed, suggesting that the QDs in a SDNA matrix did not significantly alter the binding environment of DNA molecules. Thus the SDNA thin film acts as a suitable host for QD luminophores.

The SDNA thin film, particularly that with QDAs, showed significant luminescence under UV light. The photographs in Fig. 3d show the luminescence of QDC(A)-embedded SDNA thin films at excitation wavelengths of 254 and 365 nm. The excitation-dependent emission characteristics confirm the optical quality of QD-embedded SDNA thin films. Due to the differences in QD configuration (shown in Fig. 1a,b) and shell passivation between QDC- and QDA-embedded SDNA thin films, different emissions were observed (*i.e*. brighter with QDA-embedded SDNAs than QDCs). The ZnS shell on the QDs (QDAs) prevents chemical degradation and eliminates the dangling bonds, which improves the passivation of QDAs. Consequently, QDA-embedded SDNA thin films have a higher probability of radiative relaxation of photons. Interestingly, the QDA-SDNA thin film shows the 22% PLQY compare to QDA without SDNA in aqueous phase, which has only 13%. This indicates the 70% boost of PLQY efficiency due to the high aspect-ratio of SDNA duplex which eventually contributed for better passivation of QDA surface. A previous finding also supports the fluorescence intensity increase up to the order of 3 when the virtually non-fluorescent dye complexes stabilized in DNA^31^. In contrast, QDC (CdSe core without a shell) undergo chemical degradation and dangling bonds (caused trap states), hence QDC-embedded SDNA thin films (PLQY: 1.2%) have a lower probability of radiative relaxation.

### QD concentration- and configuration-dependent optical absorbance properties of SDNA thin films

**Figure 4:**
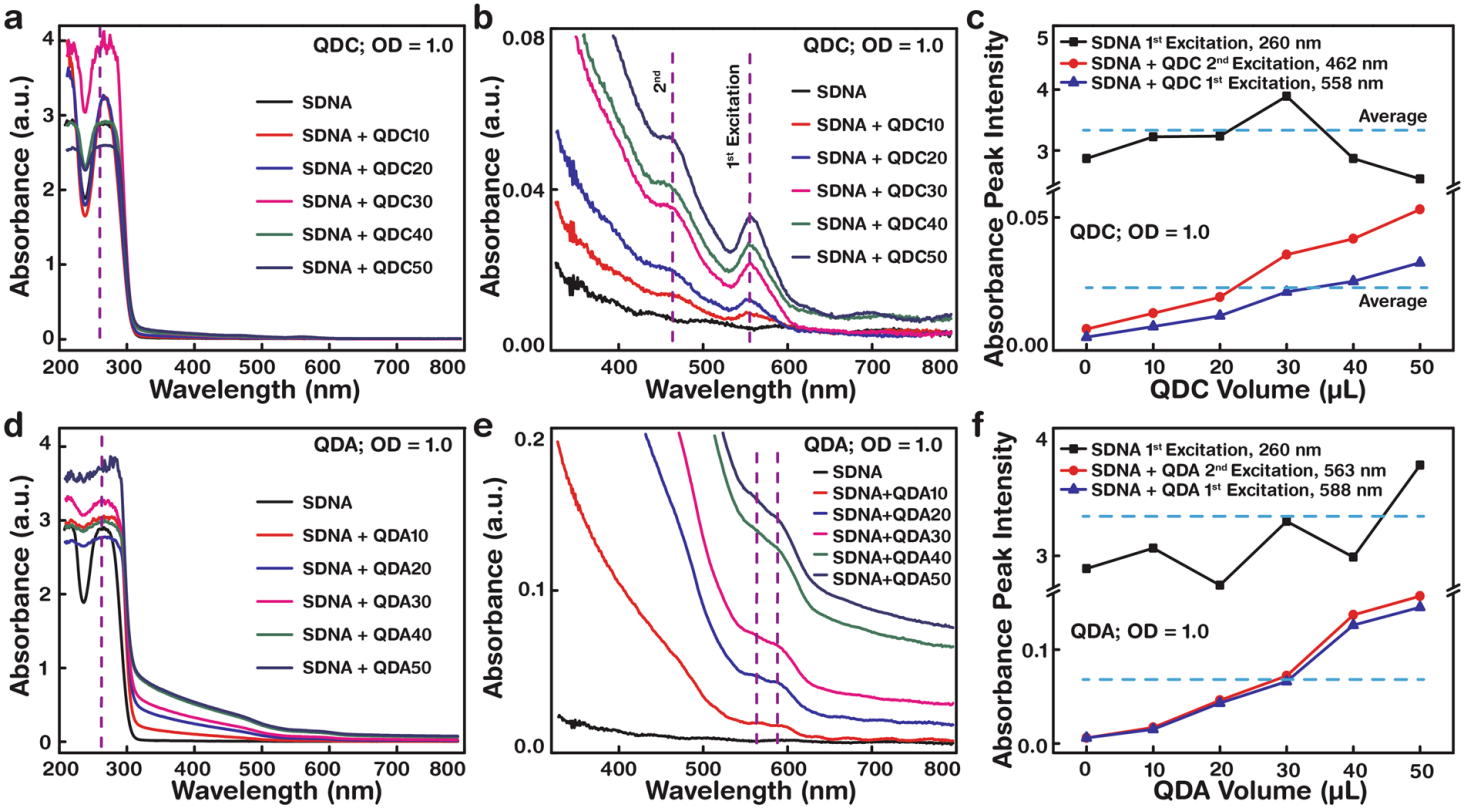
UV-vis absorption spectra of SDNA thin films with various concentrations of QDCs and QDAs at an optical density (OD) of 1.0. (**a**,**b**) Absorbance of QDC-embedded SDNA thin films (fabricated on a quartz substrate) with wavelength ranges of 200–800 and 350–800 nm, respectively. The first excitation maximum was at λ = 260 nm for the pristine SDNA thin film and 558 nm for the QDC-embedded SDNA thin film. (**c**) Absorbance peak intensity graph showing the trends of the first (λ = 558 nm) and second excitation (462 nm) maximum absorbance peak intensities (average of the first and second absorbance maximum peak intensities is 0.023 marked as a horizontal dotted line) of QDC-embedded SDNA thin films (roughly linearly proportional) and the first excitation maximum peak of pristine SDNA (average first absorbance maximum peak intensity is 3.182; roughly independent) as a function of QDC concentration (varying the volume of QDC from 0 to 50 µL). (**d**,**e**) Absorbance of QDA-embedded SDNA thin films with different wavelength ranges. The first excitation maximum was at λ = 260 nm for pristine SDNA and 588 nm for QDA-embedded SDNA. (**f**) Trends of the first (λ = 588 nm) and second excitation (563 nm) maximum absorbance peak intensities of QDA-embedded SDNA (roughly linearly proportional) as a function of QDA concentration.

DNA has a large transparency window and the maximum absorption peaks are determined by the conjugated π electrons in the phenyl rings^32^. We fabricated QD-embedded SDNA thin films on a quartz rather than a glass substrate to avoid background absorbance in the visible region (a quartz substrate has negligible absorbance in the wavelength range 200–800 nm). Absorption spectra of QD-embedded SDNA thin films were obtained by UV-vis spectrophotometer (discussed in detail in the Methods section). The absorbance maxima of pristine SDNA was found at 260 nm, as expected (dotted lines in Fig. 4a,d) and were the peak intensity maximum altered due to the QD embedment within the SDNA matrix and changes in the film thickness (Supplementary Figure S4). We controlled the net amount of QDs (with a fixed OD of 1.0) within the SDNA matrix by altering the SDNA to QD volume ratio (16:1, 16:2, 16:3, 16:4, and 16:5). Based on the absorbance analysis, the first and second (higher Stoke shift than first excitation) excitonic maxima of the QDC(A)-embedded SDNA thin films were found at 558 nm and 462 (dotted lines in Fig. 4b) (588 and 563 nm, Fig. 4e) respectively, and the first and second absorbance maximum peaks suggested the band edge transition probability of the QDC(A)-embedded SDNA thin films (electronic state transition from the ground to higher excitation states).

The absorbance increased (roughly proportionally) with increasing QDC(A) volume ratio within the SDNA matrix, which indirectly indicated the presence of a different net amount of QDC(A) within the SDNA thin film. A comparison of absorbance maximum intensities between the QDCs in the SDNA thin film (average of the first and second absorbance maximum peak intensities was 0.023; a horizontal dotted line in Fig. 4c) and SDNAs (average first absorbance maximum peak intensity was 3.182; a horizontal dotted line in Fig. 4c) yielded a ~136-fold difference, whereas maximum peak intensities between the QDAs in the SDNA (0.070; dotted line in Fig. 4f) and SDNAs (3.132) differed by ~45-fold. The absorbance difference (*ca*. threefold) between QDCs and QDAs in SDNA might be due to the presence of additional alloys and the shell configuration in QDAs. The absorbance characteristics indicated the composition of QD-embedded SDNA thin films and predicted the transition probabilities of band edges^33^.

### The photoluminescence of QDA-embedded SDNA thin films by varying the QD luminophore concentration

**Figure 5:**
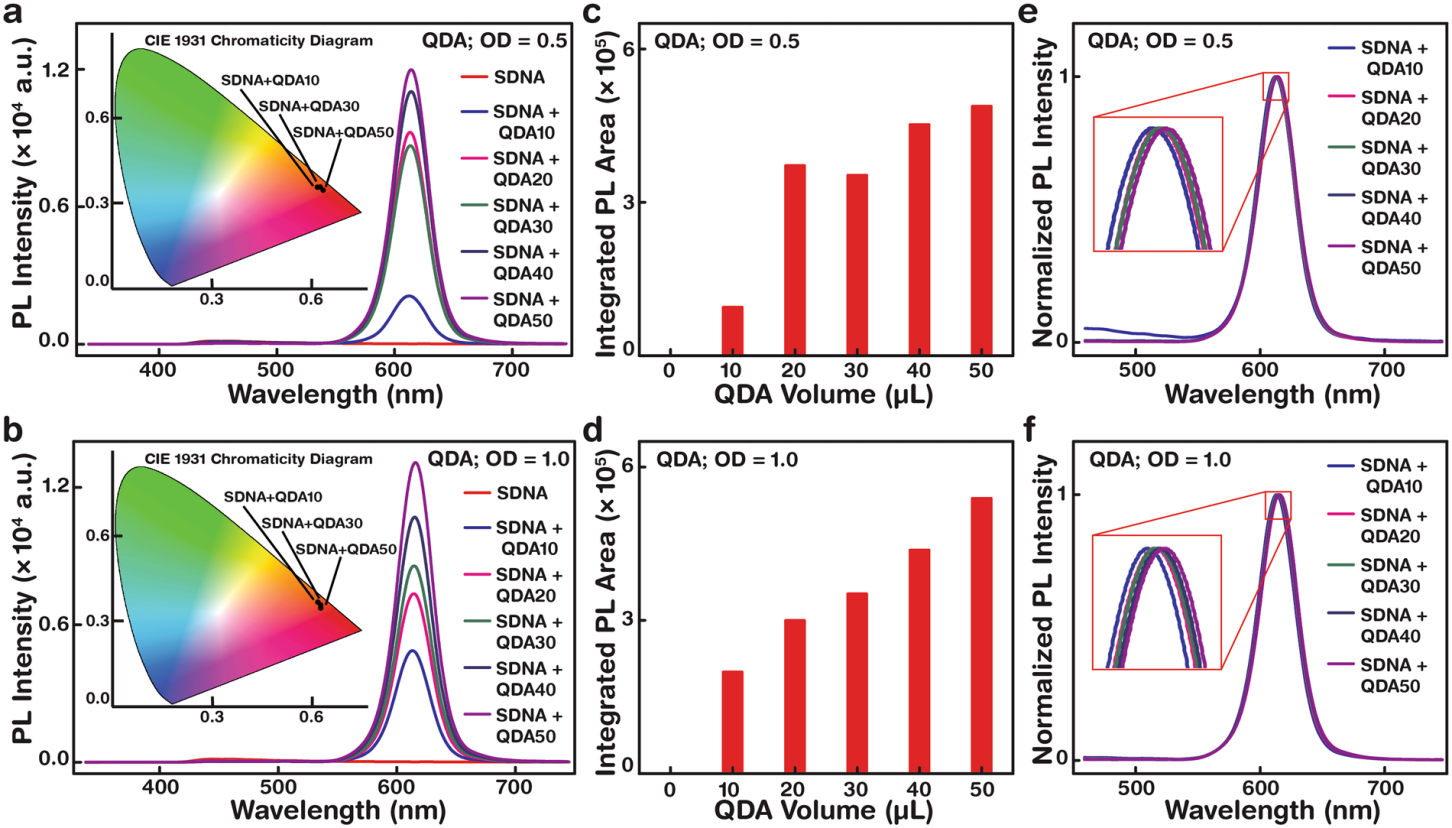
Photoluminescence characteristics of SDNA thin films with various concentrations of QDAs at optical densities (OD) of 0.5 and 1.0. (**a**) Fluorescence emissions (excited by λ = 370 nm) at OD = 0.5 were red-shifted from λ = 612 to 614 nm, hence the CIE coordinates shifted from 0.621 to 0.642 (x-axis) and from 0.371 to 0.354 (y-axis). (**b**) Similarly at OD = 1.0, fluorescence emissions were noticeably red-shifted from λ = 613 to 616 nm, consequently CIE coordinate shifts occurred from 0.638 to 0.645 and from 0.357 to 0.351 along the x- and y-axes, respectively. (**c**,**d**) Integrated emission areas of QDA-embedded SDNA thin films at OD 0.5 and 1.0, respectively. Fluorescence emission areas were roughly proportional to the QDA concentration, which was controlled by varying the volume of QDA from 0 to 50 µL. (**e**,**f**) Normalised photoluminescence emissions of QDA-embedded SDNA thin films at OD 0.5 and 1.0 showed Gaussian-like emissions due to the discrete bandgap of QDs (some overlapped peaks are magnified for clarity).

A stable luminophore improves lasing gain and photonic device stability^10, 34^ – the performance and life time of the device are determined by the non-degradation and photostability of the material. Although a photonic device comprising a Rhodamine-6G-doped DNA complex showed amplified spontaneous emission^35^, replacement of a stable and highly sensitive QD luminophore with DNA might increase the device life time and performance further, facilitating their use in biophotonics. We fabricated QDA-embedded SDNA thin films and evaluated their PL characteristics. The PL characteristics of QDA-embedded SDNA thin films fabricated using QD concentrations [QD] of OD 0.5 and 1.0 are shown in Fig. 5a,b. The PL emission area and CIE (International Commission on Illumination) coordinate shifts increased with altering of the SDNA to QDA volume ratio. The QDA-embedded SDNA thin films were excited at a wavelength of 370 nm. PL spectra for OD 0.5 (1.0) showed red-shifted emission from 612 to 614 nm (613 to 616 nm); hence the CIE coordinate colour-shifts were from 0.621 to 0.642 (0.638 to 0.645) along the x-axis and from 0.371 to 0.354 (0.357 to 0.351) along the y-axis (Supplementary Table S5).

Radiative and polarised emissions probability ‒ proportional to the integrated PL emission area – from the QDA-embedded SDNA thin films increased roughly linearly while altering the volume ratio of SDNA to QDs (Fig. 5c,d). Thus the PL emission sensitivity of QDA-embedded SDNA thin films could be easily controlled by altering the volume ratio of the QDA luminophore. SDNA functions as a superior host that can organise and stabilise the QDA luminophores in a 3D matrix and significantly reduces luminophore self-quenching. Also, SDNA interacts with the QDA luminophore surface and provides a superior passivation environment, which enhances the probability of radiative and polarised emission through the modified quantum states of QDA-SDNA interactions within the matrix. The normalised PL spectra indicated slight emission shifts and Gaussian-like narrow emission (FWHM, ~35 nm), indicating the discrete band edge emissions of QDA-embedded SDNA thin films (Fig. 5e,f). The PL emission characteristics of the QDA-embedded SDNA thin film can be implied as a photo-induced signal in photonic-based sensors and be acknowledged as a change of bio-environment in a biological transducer *via* the emission shift.

### Charge transport characteristics of QD-embedded SDNA thin films

**Figure 6:**
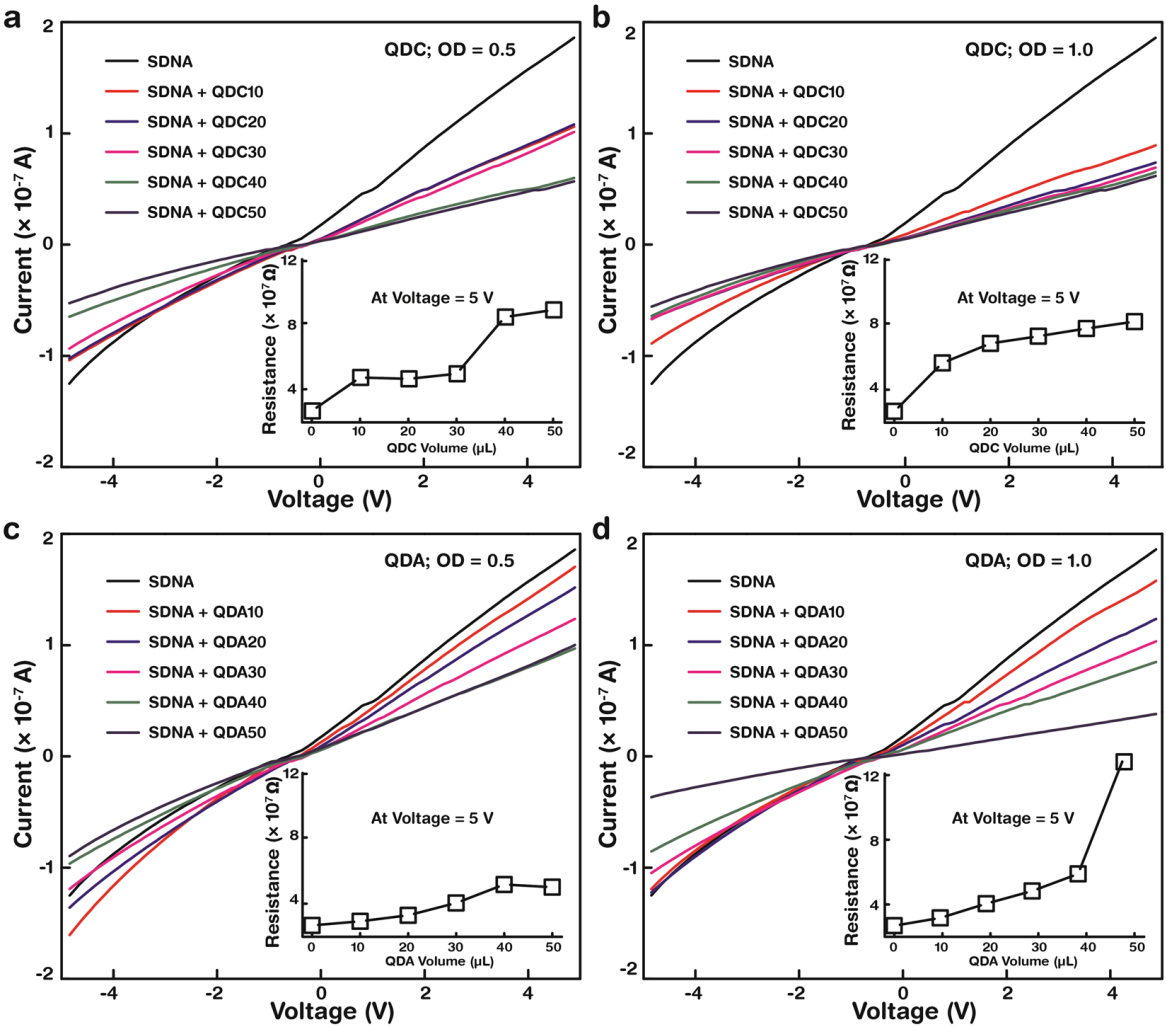
Electrical characteristics of SDNA thin films with various concentrations of QDCs and QDAs at optical densities (OD) of 0.5 and 1.0. Specific currents controlled by bias voltages from −5 to +5 V through (**a**) a QDC-embedded SDNA thin film with OD = 0.5, (**b**) a QDC-embedded SDNA with OD = 1.0, (**c**) a QDA-embedded SDNA with OD = 0.5 and (**d**) a QDA-embedded SDNA with OD = 1.0. The pristine SDNA thin film (without QDs) showed a higher current compared to the QD-embedded SDNAs (with QDs). Here, a QD served as a current-reducer within the SDNA thin film due to its semiconducting characteristic. Insets show resistance of the corresponding QD-embedded SDNA thin film at a fixed voltage, 5 V. Resistance increased with increasing QD volume from 0 to 50 µL. The overall behaviour of the resistance was roughly proportional to the QD concentration.

Current-voltage (*I-V*) measurements (discussed in detail in the Methods section) were performed to evaluate the charge carrier transport properties of QDC(A)-embedded SDNA thin films, which are essential for electronics applications. QDC(A)-embedded SDNA thin films were fabricated on a glass substrate by altering the SDNA to QDC(A) volume ratio, and silver electrodes were deposited as the probe contacts. The electrical properties were analysed using a semiconductor parameter analyser; the data was shown in Fig. 6a,b for QDC-embedded SDNA thin films and Fig. 6c,d for QDA-embedded SDNA thin films. The output *I* was obtained by the preset input *V* in the range −5 V to +5 V. Insets show resistance (*R* = *V/I*) as a function of the SDNA to QD volume ratio at a fixed *V* = 5 V. The *R* ranged from a few tens of MΩ (pristine SDNA thin film) to a few hundred (QD-embedded SDNA). The SDNA thin films without QDs showed a lower *R* (26.9 MΩ) than the QD-embedded SDNA thin films. *R* increased gradually with increasing the QDC(A) volume ratio to 87.9 MΩ (131.0 MΩ). Although QDs are semiconductors, the measured *I* decreased with increasing [QD], possibly due to the limitation of packing of QDs within the SDNA matrix, thin film’s thickness variation and the increment of interfacial barriers between the QDs and the SDNA.

## Discussion

QDs (core- and core/alloy/shell-type) ‒ synthesized by fast, simple hot-injection method and surface functionalized by mass-action principle ‒ were dispersed in organic and aqueous media, a salmon DNA thin film containing QDs was prepared, and its optoelectronic characteristics were investigated. The internal configuration and shell passivation of the QDs was extremely sensitive and responsible for its high fluorescence emission and PLQY. No chemical interaction between the QDs and SDNA molecules was evident from the FTIR absorbance bands, also the measured PLQY of QDA aqueous solution was 13% where the QDA-SNDA thin film was 22%, suggesting that the SDNA matrix could function as an efficient host for QD luminophores. UV-vis absorbance peak intensities confirmed the presence of QDs (with differentiation of a QD configuration) within the SDNA matrix, which increased with increasing [QD]. The photoluminescence of SDNA thin films with various [QD] showed narrow Gaussian-like fluorescence with a discrete bandgap. A monotonic increase in the integrated emission area and slight red-shift of the emission (confirmed by the CIE chromaticity diagram) controlled by various [QD] were observed. Current-voltage measurements of QD-embedded SDNA thin films were performed. The SDNA thin films without QDs showed lower resistance than QD-embedded SDNA thin films due to the limitation of packing of QDs within the SDNA matrix and the increment of interfacial barriers between the QDs and SDNA. QDs (stable luminophores) in a SDNA thin film template (an efficient host) have been suggested to have promise as a functionalised platform for multifunctional devices and highly sensitive sensors for bioimaging and biophotonics.

## Methods

### Core- and core/alloy/shell-type quantum dot (QD) synthesis

Cadmium oxide (CdO, 99.99%), zinc acetate (Zn(Ac), 99.9%), selenium (Se), sulphur (S), trioctylphosphine (TOP, 90%), oleic acid (OA, 90%), and 1-octadecene (ODE, 90%) were used for QD synthesis (Sigma-Aldrich, St. Louis, USA). CdO (1.60 mM), Zn(Ac) (4.00 mM) and OA 5 mL were placed in a 200 mL three-neck round-bottom flask. A temperature sensor was inserted into the flask and heated to 150 °C with stirring at 450 rpm. Nitrogen gas (N_2_) was flowed into the flask and an N_2_ atmosphere was maintained inside the flask throughout the experiment to prevent chemical oxidation. Fifteen millilitres of ODE were injected into a flask at 150 °C and the system was heated to 310 °C at a rate of 15 °C/min. At 310 °C, 3 mL of stock solution; *i.e*. Se, S + TOP (prepared by dissolving Se (0.40 mM) and S (1.33 mM) in 3 mL of TOP solvent) were injected into the system rapidly and cooled to 280 °C followed by incubation (15 min) to enable QD growth and surface reconstruction (Supplementary Figure S1). Core (CdSe)-type QDs (QDC) and core/alloy/shell (CdSe/Cd_1−x_Zn_x_Se_1−y_S_y_/ZnS)-type QD (QDA) structures were formed in the absence and presence of Zn and S, respectively (Fig. 1a).

### HRTEM/STEM/HAADF elemental mapping

QDAs dispersed in chloroform (CF) at 5 mg/mL were drop-casted on a copper mesh grid of a high-resolution transmission electron microscope (HRTEM), followed by drying for 1 h at 60 °C. The grid with QDAs was placed inside the HRTEM (Cs-Corrected/EDS/EELS, JEM ARM 200 F, JEOL Corp., Tokyo, Japan) under an ultra-vacuum for 3 h to remove residue and moisture prior to high-angle annular dark-field (HAADF) elemental mapping (Fig. 1b and Supplementary Figure S2).

### Ligand exchange of QDs for aqueous dispersion

Purified QDs dispersed in CF were placed in a vial (containing 5 mg QDs dispersed in 1 mL CF), followed by addition of 0.2 mL of MPA. The mixture (QD + CF + MPA) was vortexed for 2 min to achieve homogeneity. The MPA-treated QD residues were decanted by centrifugation for 5 min at 8,000 rpm. After removal of excess solvent, an equal amount of buffer (ammonia) was added as a co-stabilising agent. Finally, the QDs were dispersed in deionised (DI) water (Fig. 1c).

### AFM imaging of QDs

For AFM imaging, 5 µL of QDs dispersed in the DI water were dropped on a mica substrate attached to a metal puck using instant glue. Then, 30 µL of DI water were pipetted onto the mica and a further 30 µL were dispensed into the SiN AFM tip (NP-S10, Veeco Inc., NY, USA). AFM images were obtained using a Multimode Nanoscope III (Veeco Inc., NY, USA) in liquid-tapping mode (Fig. 1c and Supplementary Figure S3).

### Preparation of salmon DNA (SDNA) solution

To prepare the SDNA solution (Chitose Institute of Science and Technology, Hokkaido, Japan), 0.1 g of purified SDNA are dissolved in 10 mL of DI water (the resulting solution has a concentration of one weight-percentage (wt.-%) SDNA in DI water) followed by the magnetic stirring (with 1,000 rpm for 10 h at room temperature) to achieve a homogeneous mixture of SDNA in solvent (Fig. 3a).

### Preparation of QD-embedded SDNA solution

SDNA solution (1 wt.-%) with a volume of 160 μL was pipetted into an Eppendorf test-tube. Two different QD concentrations ([QD], measured using an UV-vis absorption spectrometer), optical densities (OD) of 0.5 and 1.0 (which correspond to the first excitation maximum peak in the absorbance spectra) were prepared. QD solutions of OD = 0.5 or 1.0 (0, 10, 20, 30, 40, and 50 μL) were added to a test-tube containing SDNA solution, followed by vortex mixing for 5 min and then incubation for 24 h at room temperature to obtain a homogeneous QD-embedded SDNA solution (Fig. 3a and Supplementary Table S2).

### Fabrication and thickness measurement of a QD-embedded SDNA thin film on a given substrate

Glass (for PL and *I–V*) and quartz (for absorbance and FTIR) with a size of 5 × 5 mm^2^ were treated with O_2_ plasma for surface modification. The O_2_ plasma treatment introduced silanol groups to the substrate. This changed the net charge from neutral to negative, resulting in a change from a hydrophobic to a hydrophilic substrate. An O_2_ plasma cleaner (Plasma Processing System, CUTE–1MP/R, Gyeonggi, Korea) was used in this study. The O_2_ plasma process was performed under the following conditions: 50 W power, 5 × 10^−2^ Torr base pressure, 47 SCCM oxygen flow rate, 7.8 × 10^−1^ Torr working oxygen pressure, and plasma generation time of 5 min for glass and 10 min for quartz substrates. Following O_2_ plasma exposure, 20 μL of QD-embedded SDNA solution were drop-casted and allowed to dry for a day to fabricate a QD-embedded SDNA thin film (Fig. 3a).

The thickness of pristine SDNA and QD-embedded SDNA thin films was measured using a surface profilometer (Alpha-Step D-500, KLA-Tencor Corp., CA, USA) and a field emission scanning electron microscope (FE-SEM) (JSM 7401 F, JEOL Corp., Tokyo, Japan). The measured average thickness of the thin films was 4.5 µm for SDNA, 4.3 µm for a QD-embedded SDNA thin film with a 10 µL QD volume at a given SDNA volume of 160 µL and 1.6 µm for QD-embedded SDNA with a 50 µL QD volume (Supplementary Figure S4).

### Fourier transform infrared (FTIR) spectroscopy measurement

The FTIR spectra (the TENSOR 27 spectrometer with a detector MIR_ATR (ZnSe), Bruker Inc., MA, USA) of the QDs in solution (Fig. 2a) and a QD-embedded SDNA thin film (Fig. 3b,–c) were obtained in the range 3,600 to 600 cm^−1^. Thirty-two scans were co-added and averaged at a resolution of 4 cm^−1^. The data in the FTIR spectra used in the present study were analysed by subtracting the background spectrum produced by a bare substrate (Supplementary Tables S1, S4).

### UV-vis absorption measurement

A spectrophotometer (Cary 5 G, Varian, CA, USA) was used to conduct optical absorbance measurements of QDs in solution (Fig. 2b) and a QD-embedded SDNA thin film (Fig. 4) obtained in the visible and UV regions in the range 800 to 200 nm. The spectrophotometer was equipped with two light sources: a deuterium arc lamp (for near-infrared and visible) and a quartz W-halogen lamp (for UV). The instrument has two detectors: a cooled PbS detector (for near-infrared) and a photomultiplier tube (for visible and UV). The spectrophotometer measures the frequency-dependent light intensity passing either through a vacuum or through the sample.

### Absolute photoluminescence quantum yield (PLQY) measurement

The PLQY of QD solution (ODs of ~0.07 and 0.5) and QDC(A)-embedded SDNA thin films were measured by absolute method using an integration sphere (C9920-20, Hamamatsu Photonics, Japan). The PL spectra were recorded in the wavelength range of 200–950 nm at the fixed excitation wavelength of 350 nm and corrected based on system response (Supplementary Table S3).

### Photoluminescence (PL) measurement

The PL excitation and emission spectra of the QDs in solution (Fig. 2b,c) and a QD-embedded SDNA thin film (Fig. 5) were measured at room temperature using a Xe-arc lamp equipped with a flurometer (FS-2, Scinco, Seoul, Korea) with a power of 25W. The excitation spectra were obtained at a fixed emission wavelength of 615 nm, while the emission spectra were measured by exciting the samples at a fixed excitation wavelength of 370 nm.

### Current‒voltage (*I*‒*V*) measurement

The electrical property of a QD-embedded SDNA thin film on glass was determined using a semiconductor parameter analyser (4200–SCS, Keithley Instruments Inc., OH, USA) in the dark at room temperature. Silver pastes (as metal electrodes with a channel gap distance of ~1 mm) were placed on a QD-embedded SDNA thin film prior to measurement (Fig. 6).

## Electronic supplementary material

Supplementary Information
