# Supplementary material for: Luminophore Configuration and Concentration-Dependent Optoelectronic Characteristics of a Quantum Dot-Embedded DNA Hybrid Thin film
Source: Sci Rep. 2017 Sep 14;7:11567. doi: 10.1038/s41598-017-11797-7 (PMC5599517; doi:10.1038/s41598-017-11797-7)
Supplement: Supplementary file 1 — Supplementary Information [file 41598_2017_11797_MOESM1_ESM.pdf]

Supplementary Information

Luminophore Configuration and Concentration-Dependent Optoelectronic Characteristics of a Quantum Dot-Embedded DNA Hybrid Thin film

Velu Arasu[1]*, Sreekantha Reddy Dugasani[1,2]*, Mallikarjuna Reddy Kesama[2], Ho Kyoon Chung[1] & Sung Ha Park[1,2]

[1] Sungkyunkwan Advanced Institute of Nanotechnology (SAINT), Sungkyunkwan University, Suwon 16419, Korea

[2] Department of Physics, Sungkyunkwan University, Suwon 16419, Korea

*These authors contributed equally to this work.

Correspondence and requests for materials should be addressed to H.K.C. (email: hokchung@skku.edu,) or S.H.P. (email: sunghapark@skku.edu).

Figure S1. Schematics of QDC and QDA synthesis.

Figure S2. Quantitative analysis of QD weight and atomic percentage measured by an energy dispersive spectrometer (EDS).

Figure S3. Size measurement of a QDA *via* a representative AFM image.

Figure S4. Thickness measurement of pristine SDNA and the QD-embedded SDNA thin films by a surface profilometer (SP) and a field emission scanning electron microscope (FE-SEM).

Table S1. FTIR spectral band position and band assignment of QD-OA (organic) and QD-MPA (aqueous) colloidal samples.

Table S2. Sample preparation and naming for a QD-embedded SDNA thin film.

Table S3. Absolute PLQY of QD solutions and QD-embedded SDNA thin films.

Table S4. FTIR spectral band position and band assignment of a QD-embedded SDNA thin film.

Table S5. CIE coordinate spectral shift and integrated emission area of a QD-embedded SDNA thin film.

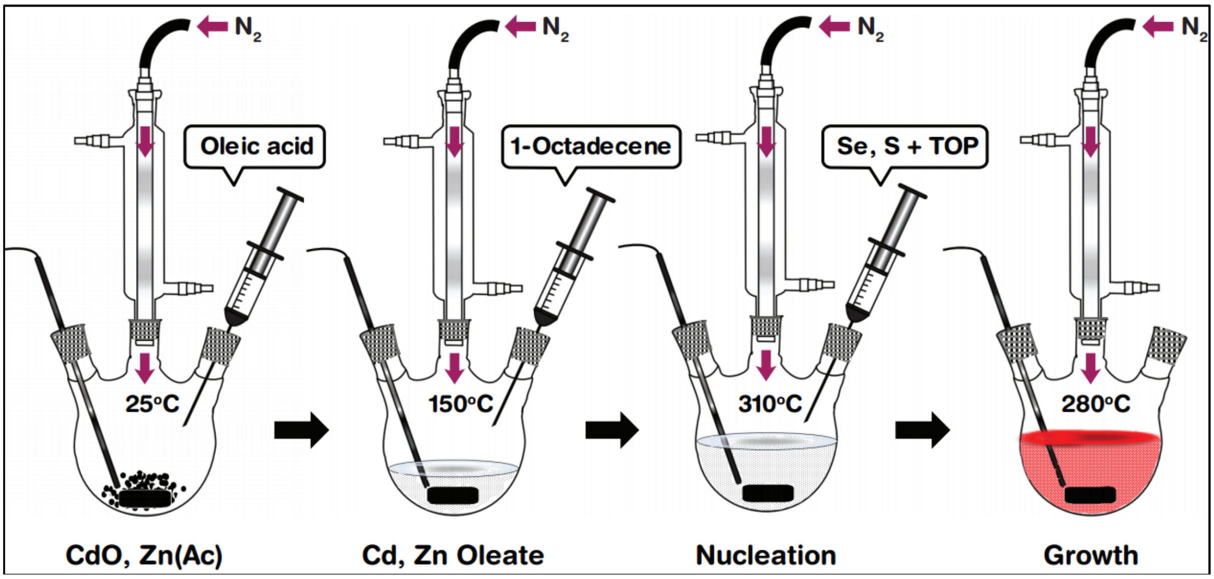

Figure S1 | Schematics of QDC and QDA synthesis. We adopted the one-pot synthesis scheme using a hot-injection method with an appropriate gradient chemical composition (*i.e.* difference in reaction rate (up to 10-fold) between the Se with Cd oleate solution and S with Zn), which enables tuning of QD configuration[1]. CdSe core-type QDs (QDC) and CdSe/$Cd_{1-x}Zn_xSe_{1-y}S_y$/ZnS core/alloy/shell-type QD (QDA) configurations were formed in the absence and presence of Zn and S, respectively. The one-pot synthesis technique yields core/alloy/shell-type QDs with a distinguishable configuration (CdSe/$Cd_{1-x}Zn_xSe_{1-y}S_y$/ZnS) for high fluorescence emission.

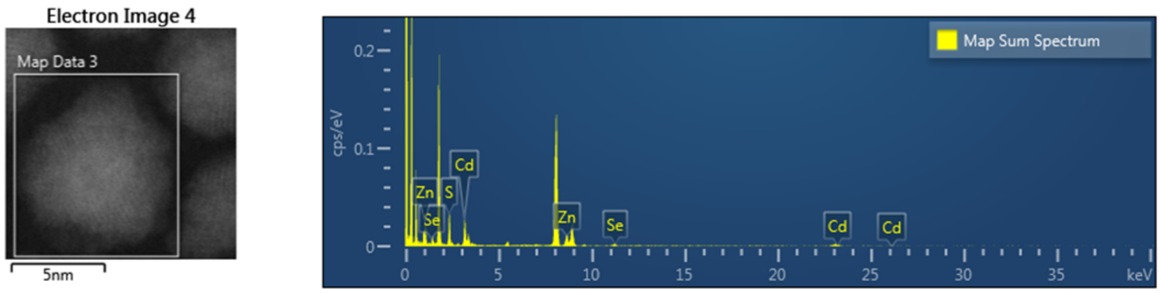

Element	Line Type	k factor	Absorption Correction	Wt%	Wt% Sigma	Atomic %
S	K series	1.03107	0.96	21.86	2.00	45.83
Zn	K series	1.07155	0.92	11.68	1.69	12.01
Se	L series	1.74238	1.04	9.59	2.34	8.16
Cd	L series	1.92121	0.94	56.87	2.98	34.00
Total:				100.00		100.00

Figure S2 | Quantitative analysis of QD weight and atomic percentages measured by an energy dispersive spectrometer (EDS). Energy dispersive spectrum confirmed the presence of specific elements within the QDs through backscattered electron energy; and the weight percentage (atomic percentage) of S, Zn, Se, and Cd were 21.86 (45.83), 11.68 (12.01), 9.59 (8.16), and 56.87 (34.00), respectively.

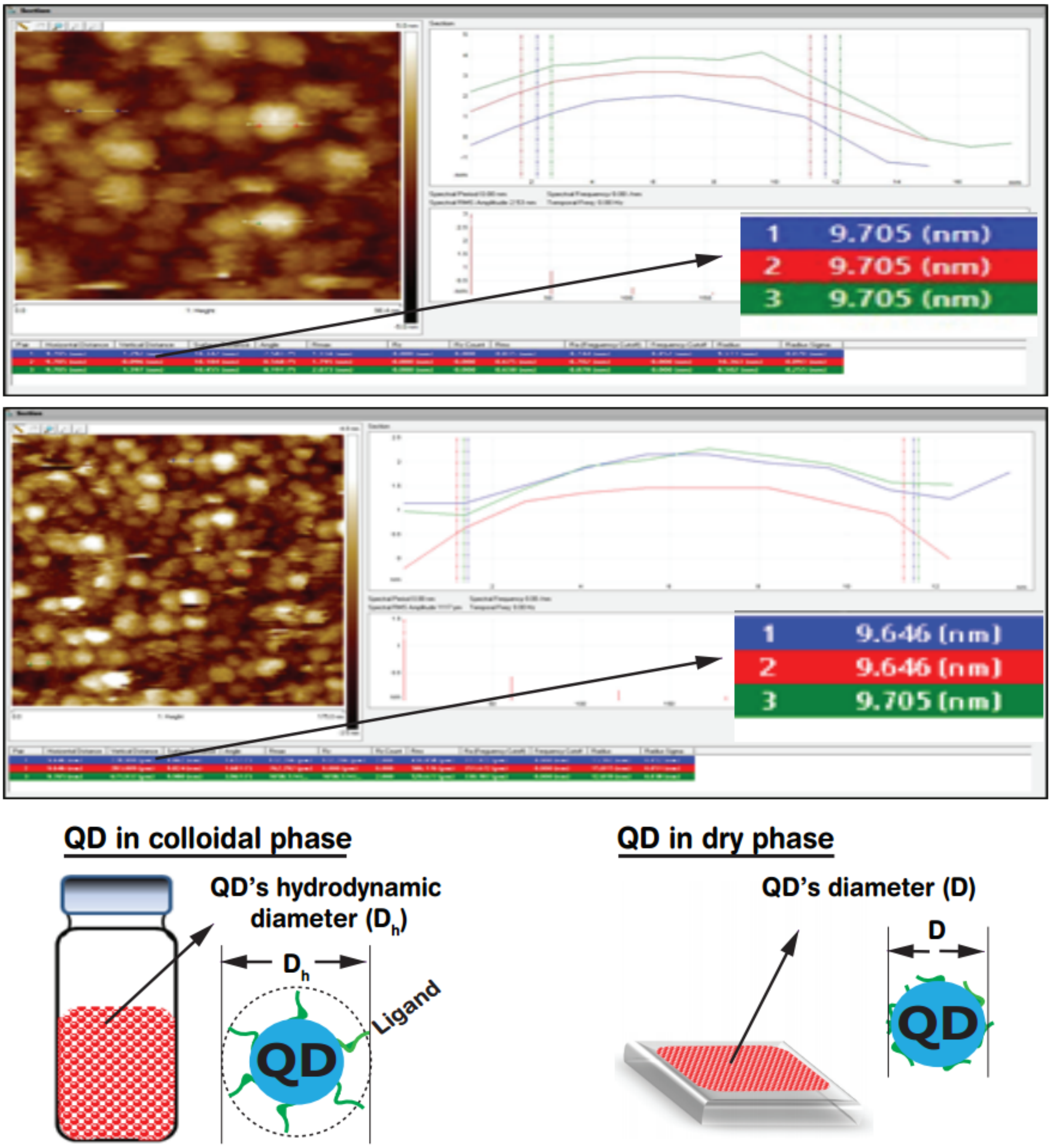

Figure S3 | Size measurement of a QDA *via* a representative AFM image. Representative AFM image (scan sizes of ~100 × 100 nm^2 (top) and ~200 × 200 nm^2 (bottom); flattened) obtained by fluid tapping mode shows an average QDA hydrodynamic diameter of 9.68 ± 0.2 nm. Also the schematics of QDs diameter while in colloidal (hydrodynamic diameter) and dry phase. Hydrodynamic diameter is slightly extended than the actual particle diameter due to the inclusion of the ligands (electrostatically and hydrodynamically dominant in colloidal state) length as well.

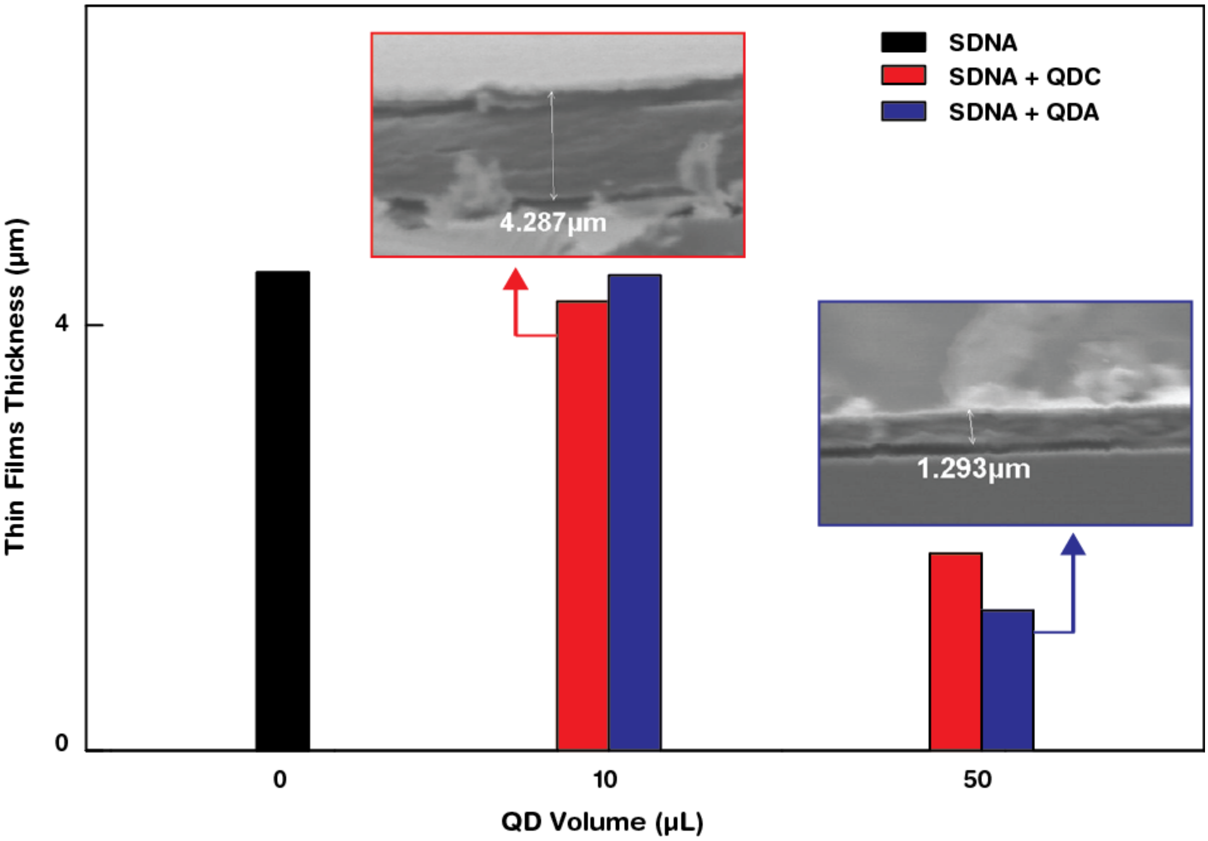

Figure S4 | Thickness of pristine SDNA and QD-embedded SDNA thin films by a surface profilometer (SP) and a field emission scanning electron microscope (FE-SEM). Thin-film thickness was measured by a surface profilometer (equipped with an Alpha-Step IQ software interface for analysing the cross-sectional profile) and an FE-SEM (equipped with a rotational sample stage for measuring the cross-sectional image). The average thickness of the thin films was 4.495 μm for pristine SDNA, 4.343 μm for QD-embedded SDNA (4.219 μm for QDC and 4.468 for QDA) with a 10 μL QD volume in 160 μL SDNA and 1.587 μm for a QD-embedded SDNA (1.855 μm for QDC and 1.318 for QDA) with a 50 μL QD volume. Representative FE-SEM images are shown in the insets, and were in good agreement with the surface profilometer cross-sectional measurements (± 2% differences). In the FE-SEM measurement, the thin film was coated with a platinum conductive layer (~10 nm) by sputtering to reduce thermal damage and improve secondary electron signals.

From the average thickness values, the estimated thickness of a QD-embedded SDNA thin film controlled by the QD volume can be written (with the assumption of linearity between the SDNA thin film thickness and volume of the QD) as $T = T\left(V_{QD}\right) = -\,a\,V_{QD} + 4.3435$, where T, a, and V_{QD} are the thin film thickness (μm), a constant (0.069 m/L, slope magnitude = $\frac{|1.5865\ - 4.3435\ |}{50-10}\left(\frac{\mathrm{m}}{\mathrm{L}}\right)$) and the QD volume (μL), respectively.

Table S1 | FTIR spectral band position and band assignment of QD-OA (organic) and QD-MPA (aqueous) colloidal samples.

No.	Band Position (cm^{-1})	Band Assignment[2]
1	770	C-H bending; Organic fingerprint present in chloroform
2	1400	Carboxylate symmetric stretching vibrations of MPA ligand attachment on the QDC-MPA colloidal samples
3	1750	Carboxylate asymmetric stretching vibrations of MPA ligand attachment on the QDA-MPA colloidal samples
4	2900	C-H stretching vibrations of OA ligand attachment on the QD-OA colloidal samples
5	3050	N-H bond stretch; Presence of Ammonia co-solvent
6	3140	CO-OH; Carboxylic functional group absorption band

Table S2 | Sample preparation and naming of QD-embedded SDNA thin films.

No.	Volume of SDNA	Volume of QD (QDC = QD Core; QDA = QD Alloy)	Sample Name
0	160 µL	-	SDNA
1	160 µL	QDC / 10 µL	SDNA + QDC10
2	160 µL	QDC / 20 µL	SDNA + QDC20
3	160 µL	QDC / 30 µL	SDNA + QDC30
4	160 µL	QDC / 40 µL	SDNA + QDC40
5	160 µL	QDC / 50 µL	SDNA + QDC50
6	160 µL	QDA / 10 µL	SDNA + QDA10
7	160 µL	QDA / 20 µL	SDNA + QDA20
8	160 µL	QDA / 30 µL	SDNA + QDA30
9	160 µL	QDA / 40 µL	SDNA + QDA40
10	160 µL	QDA / 50 µL	SDNA + QDA50

Table S3 | Absolute PLQY of QD solutions and QD-embedded SDNA thin films.

QDs	Optical density [QD] at 1st excitation	PLQY in liquid phase (%)		DNA:QDs Concentration (µL)	PLQY in solid phase (%)
		In chloroform (before ligand exchange)	In DI water (after ligand exchange)		QDC(A)-SDNA thin films
QDC	0.07	7.0	2.0	160:10	1.2
				160:50	0.5
	0.5	3.2	0.5	160:10	1.1
				160:50	0.4
QDA	0.07	61.0	13.0	160:10	22.0
				160:50	18.4
	0.5	57.1	10.7	160:10	18.0
				160:50	16.5

Table S4 | FTIR spectral band position and band assignment of QD-embedded SDNA thin films.

No.	Band Position (cm^{-1})	Band Assignment[3]
1	780	Sugar phosphate vibration
2	828	Deoxyribose phosphate
3	895	Adenine–Thymine base pairs
4	960	C–C and C–O of deoxyribose skeletal motion
5	1010	P–O or C–O stretching
6	1055	C–O deoxyribose stretching
7	1083	Phosphate symmetric stretching
8	1230	Phosphate antisymmetric stretching
9	1372	Cytosine and Guanine
10	1416	Cytosine and Guanine
11	1488	Cytosine (in-plane vibration)
12	1604	Adenine (C7=N stretching)
13	1652	Thymine (C2=O stretching)
14	1693	Guanine (C=O stretching)
15	3357	OH stretching

Table S5 | CIE coordinate spectral shift and integrated emission area of QD-embedded SDNA thin films. CIE coordinates calculated using the Colour Quality Scale (CQS) software and integrated emission area measured under the curve of the x-axis with wavelength in the range 370-750 nm.

No.	Sample<br>*QDA (OD=0.5)*	CIE x	CIE y	Integrated<br>Emission Area (× 10^5)
1	SDNA + QDA10	0.621	0.371	0.943
2	SDNA + QDA20	0.640	0.355	3.716
3	SDNA + QDA30	0.639	0.356	3.527
4	SDNA + QDA40	0.641	0.355	4.520
5	SDNA + QDC50	0.642	0.354	4.879
	QDA (OD=1.0)			
6	SDNA + QDA10	0.638	0.357	1.987
7	SDNA + QDA20	0.642	0.354	2.995
8	SDNA + QDA30	0.643	0.353	3.518
9	SDNA + QDA40	0.644	0.352	4.375
10	SDNA + QDA50	0.645	0.351	5.386

References

1. Bae, W. K., Char, K., Hur, H. & Lee, S. Single-Step Synthesis of Quantum Dots with Chemical Composition Gradients. *Chem. Mater*. **20,** 531–539 (2008).
2. Jeong, K. S. *et al.* Enhanced Mobility-Lifetime Products in PbS Colloidal Quantum Dot Photovoltaics. *ACS Nano.* **6,** 89-99 (2012).
3. Gnapareddy, B. *et al.* Chemical and Physical Characteristics of Doxorubicin Hydrochloride Drug-Doped Salmon DNA Thin Films. *Sci. Rep.* **5,** 12722-8 (2015).
